# 3-Acetyl-2,4-di­methyl­quinolin-1-ium chloride

**DOI:** 10.1107/S160053681301684X

**Published:** 2013-06-22

**Authors:** R. Prasath, P. Bhavana, Seik Weng Ng, Edward R. T. Tiekink

**Affiliations:** a Department of Chemistry, BITS, Pilani - K. K. Birla Goa Campus, Goa 403 726, India; b Department of Chemistry, University of Malaya, 50603 Kuala Lumpur, Malaysia; c Chemistry Department, Faculty of Science, King Abdulaziz University, PO Box 80203 Jeddah, Saudi Arabia

## Abstract

In the title salt, C_13_H_14_NO^+^·Cl^−^, the dihedral angle between the fused ring system (r.m.s. deviation = 0.039 Å) and the attached aldehyde group is 75.27 (16)°. In the crystal, the cation and anion are linked by an N—H⋯Cl hydrogen bond and the resulting pairs are connected into four-ion aggregates by π–π inter­actions between the C_6_ and pyridinium rings [3.6450 (9) Å] of inversion-related quinolinium residues.

## Related literature

For background details and biological applications of quinoline and quinoline chalcones, see: Joshi *et al.* (2011 ▶); Prasath *et al.* (2013*a* ▶). For a related structure, see: Prasath *et al.* (2013*b* ▶).
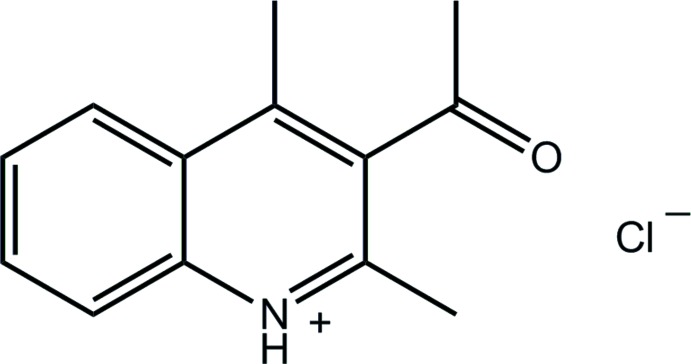


## Experimental

### 

#### Crystal data

C_13_H_14_NO^+^·Cl^−^
*M* *_r_* = 235.70
Orthorhombic, 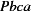

*a* = 12.8221 (6) Å
*b* = 10.7281 (4) Å
*c* = 16.3785 (6) Å
*V* = 2252.97 (16) Å^3^
*Z* = 8
Mo *K*α radiation
μ = 0.32 mm^−1^
*T* = 100 K
0.50 × 0.40 × 0.30 mm

#### Data collection

Agilent SuperNova Dual diffractometer with an Atlas detector
Absorption correction: multi-scan (*CrysAlis PRO*; Agilent, 2013 ▶) *T*_min_ = 0.956, *T*_max_ = 1.000
8404 measured reflections
2597 independent reflections
2207 reflections with *I* > 2σ(*I*)
*R*_int_ = 0.030

#### Refinement

*R*[*F* ^2^ > 2σ(*F* ^2^)] = 0.035
*wR*(*F* ^2^) = 0.099
*S* = 1.04
2597 reflections
152 parameters
H atoms treated by a mixture of independent and constrained refinement
Δρ_max_ = 0.29 e Å^−3^
Δρ_min_ = −0.29 e Å^−3^

### 

Data collection: *CrysAlis PRO* (Agilent, 2013 ▶); cell refinement: *CrysAlis PRO*; data reduction: *CrysAlis PRO*; program(s) used to solve structure: *SHELXS97* (Sheldrick, 2008 ▶); program(s) used to refine structure: *SHELXL97* (Sheldrick, 2008 ▶); molecular graphics: *ORTEP-3 for Windows* (Farrugia, 2012 ▶) and *DIAMOND* (Brandenburg, 2006 ▶); software used to prepare material for publication: *publCIF* (Westrip, 2010 ▶).

## Supplementary Material

Crystal structure: contains datablock(s) global, I. DOI: 10.1107/S160053681301684X/hb7097sup1.cif

Structure factors: contains datablock(s) I. DOI: 10.1107/S160053681301684X/hb7097Isup2.hkl

Supplementary material file. DOI: 10.1107/S160053681301684X/hb7097Isup3.cml

Additional supplementary materials:  crystallographic information; 3D view; checkCIF report

## Figures and Tables

**Table 1:** Hydrogen-bond geometry (Å, °)

*D*—H⋯*A*	*D*—H	H⋯*A*	*D*⋯*A*	*D*—H⋯*A*
N1—H1⋯Cl1	0.91 (2)	2.13 (2)	3.0374 (13)	175.4 (17)

**Fig. 1.**
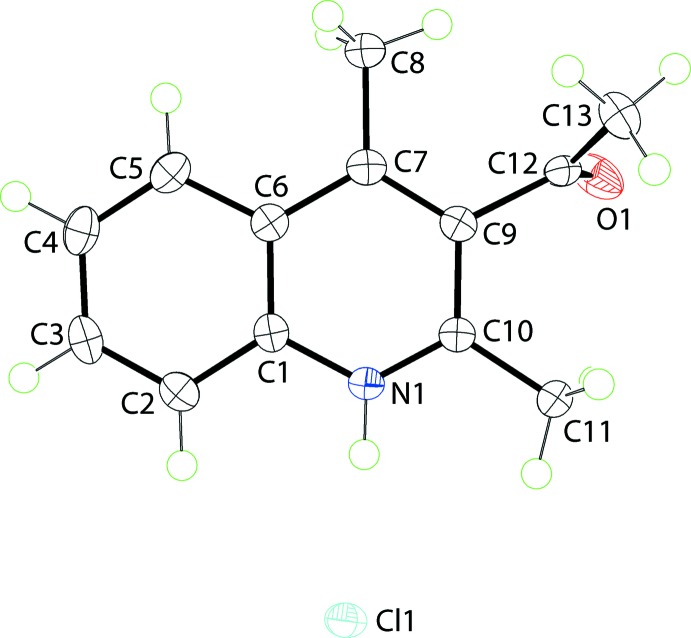
The molecular structures of the ions in (I) showing displacement ellipsoids at the 70% probability level.

**Fig. 2.**
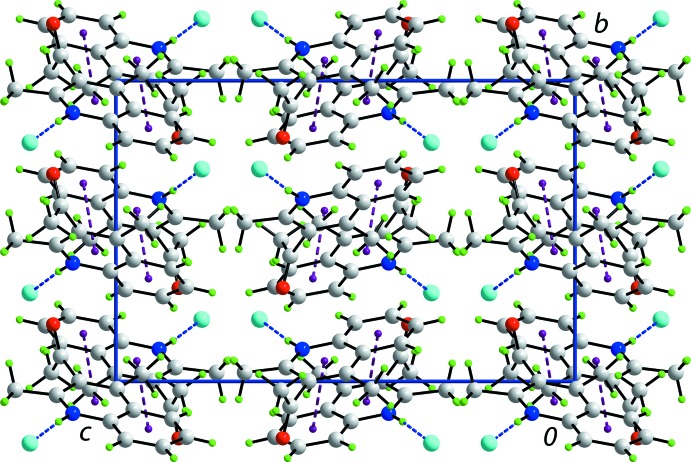
A view in projection down the a axis of the unit-cell contents of (I). The N—H···Cl and π—π interactions are shown as blue and purple dashed lines, respectively.

## supplementary crystallographic information

### Comment

Nitrogen-containing heterocyclic analogues are found to be valuable intermediates in organic synthesis and exhibit a multitude of photophysical properties. In particular, quinoline analogues have received significant attention owing to their bio-activity such as anti-bacterial, anti-fungal, anti-malarial and anti-cancer activities (Prasath *et al.*, 2013*a*; Joshi *et al.*, 2011). As a continuation of structural studies in this area (Prasath *et al.*, 2013*b*), the title salt, (I), was investigated.

The fused-ring system of the cation in (I), Fig. 1, is almost planar with the r.m.s. deviation of the fitted atoms being 0.039 Å; maximum deviations are 0.051 (1) Å for the C3 atom and -0.044 (2) Å for the C5 atom. The aldehyde group is almost perpendicular to this plane, forming a C10—C9—C12—O1 torsion angle of 73.64 (18)°.

In the crystal, ions are linked by a N—H···Cl hydrogen bond, Table 1, and connected into four-ion aggregates by π—π interactions between the C_6_ and pyridinium rings [inter-centorid distance 3.6450 (9) Å for symmetry operation 1 - *x*, 1 - *y*, 1 - *z*] of centrosymmetrically related quinolinyl residues, Fig. 2. These pack with no specific interactions between them.

### Experimental

A mixture of 2-aminoacetophenone (0.68 g, 0.005 *M*), acetylacetone (0.5 g, 0.005 *M*) and 1 N HCl (20 ml) was stirred at 363 K for 45 minutes. To the resulting mixture, chloroform (20 ml) was added and the organic layer was passed through anhydrous Na_2_SO_4_. Re-crystallization was by slow evaporation of chloroform solution of (I) which yielded yellow blocks. *M*.pt. 393–395 K. Yield: 90%.

### Refinement

The C-bound H atoms were geometrically placed (C—H = 0.95–0.98 Å) and refined as riding with *U_iso_*(H) = 1.2–1.5*U_eq_*(C). The N-bound H atom was refined freely.

### Crystal data

**Table:**

C_13_H_14_NO^+^·Cl^−^	*F*(000) = 992
*M**_r_* = 235.70	*D*_x_ = 1.390 Mg m^−^^3^
Orthorhombic, *P**b**c**a*	Mo *K*α radiation, λ = 0.71073 Å
Hall symbol: -P 2ac 2ab	Cell parameters from 3252 reflections
*a* = 12.8221 (6) Å	θ = 3.0–27.5°
*b* = 10.7281 (4) Å	µ = 0.32 mm^−^^1^
*c* = 16.3785 (6) Å	*T* = 100 K
*V* = 2252.97 (16) Å^3^	Block, yellow
*Z* = 8	0.50 × 0.40 × 0.30 mm

### Data collection

**Table:**

Agilent SuperNova Dual diffractometer with an Atlas detector	2597 independent reflections
Radiation source: SuperNova (Mo) X-ray Source	2207 reflections with *I* > 2σ(*I*)
Mirror monochromator	*R*_int_ = 0.030
Detector resolution: 10.4041 pixels mm^-1^	θ_max_ = 27.6°, θ_min_ = 3.0°
ω scan	*h* = −11→16
Absorption correction: multi-scan (*CrysAlis PRO*; Agilent, 2013)	*k* = −13→10
*T*_min_ = 0.956, *T*_max_ = 1.000	*l* = −19→21
8404 measured reflections	

### Refinement

**Table:**

Refinement on *F*^2^	Primary atom site location: structure-invariant direct methods
Least-squares matrix: full	Secondary atom site location: difference Fourier map
*R*[*F*^2^ > 2σ(*F*^2^)] = 0.035	Hydrogen site location: inferred from neighbouring sites
*wR*(*F*^2^) = 0.099	H atoms treated by a mixture of independent and constrained refinement
*S* = 1.04	*w* = 1/[σ^2^(*F*_o_^2^) + (0.0514*P*)^2^ + 0.7427*P*] where *P* = (*F*_o_^2^ + 2*F*_c_^2^)/3
2597 reflections	(Δ/σ)_max_ < 0.001
152 parameters	Δρ_max_ = 0.29 e Å^−^^3^
0 restraints	Δρ_min_ = −0.29 e Å^−^^3^

### Special details

**Table:**

Geometry. All e.s.d.'s (except the e.s.d. in the dihedral angle between two l.s. planes) are estimated using the full covariance matrix. The cell e.s.d.'s are taken into account individually in the estimation of e.s.d.'s in distances, angles and torsion angles; correlations between e.s.d.'s in cell parameters are only used when they are defined by crystal symmetry. An approximate (isotropic) treatment of cell e.s.d.'s is used for estimating e.s.d.'s involving l.s. planes.
Refinement. Refinement of *F*^2^ against ALL reflections. The weighted *R*-factor *wR* and goodness of fit *S* are based on *F*^2^, conventional *R*-factors *R* are based on *F*, with *F* set to zero for negative *F*^2^. The threshold expression of *F*^2^ > σ(*F*^2^) is used only for calculating *R*-factors(gt) *etc*. and is not relevant to the choice of reflections for refinement. *R*-factors based on *F*^2^ are statistically about twice as large as those based on *F*, and *R*- factors based on ALL data will be even larger.

### Fractional atomic coordinates and isotropic or equivalent isotropic displacement parameters (Å^2^)

**Table:**

	*x*	*y*	*z*	*U*_iso_*/*U*_eq_	
Cl1	0.38171 (3)	0.29410 (3)	0.31279 (2)	0.01712 (13)	
O1	0.78490 (9)	0.68933 (10)	0.36335 (7)	0.0257 (3)	
N1	0.56888 (10)	0.39336 (11)	0.40603 (7)	0.0127 (3)	
H1	0.5145 (17)	0.3655 (19)	0.3755 (12)	0.035 (5)*	
C1	0.56522 (12)	0.36700 (12)	0.48826 (8)	0.0130 (3)	
C2	0.47740 (12)	0.30581 (13)	0.52035 (8)	0.0156 (3)	
H2	0.4216	0.2811	0.4858	0.019*	
C3	0.47406 (13)	0.28251 (13)	0.60294 (9)	0.0177 (3)	
H3	0.4141	0.2441	0.6260	0.021*	
C4	0.55806 (13)	0.31479 (13)	0.65333 (9)	0.0185 (3)	
H4	0.5555	0.2950	0.7098	0.022*	
C5	0.64371 (13)	0.37435 (14)	0.62260 (8)	0.0174 (3)	
H5	0.6999	0.3956	0.6577	0.021*	
C6	0.64893 (12)	0.40465 (13)	0.53813 (8)	0.0140 (3)	
C7	0.73191 (11)	0.47481 (13)	0.50274 (8)	0.0139 (3)	
C8	0.81628 (12)	0.52547 (14)	0.55698 (9)	0.0192 (3)	
H8A	0.8670	0.5715	0.5239	0.029*	
H8B	0.8515	0.4564	0.5848	0.029*	
H8C	0.7855	0.5815	0.5976	0.029*	
C9	0.72892 (11)	0.49922 (13)	0.41992 (8)	0.0134 (3)	
C10	0.64623 (11)	0.45412 (12)	0.37091 (8)	0.0125 (3)	
C11	0.64361 (12)	0.47075 (13)	0.28065 (8)	0.0159 (3)	
H11A	0.5797	0.4334	0.2587	0.024*	
H11B	0.7045	0.4300	0.2562	0.024*	
H11C	0.6449	0.5599	0.2676	0.024*	
C12	0.80915 (12)	0.58304 (13)	0.37971 (8)	0.0155 (3)	
C13	0.91308 (13)	0.52994 (14)	0.35969 (9)	0.0205 (3)	
H13A	0.9622	0.5978	0.3485	0.031*	
H13B	0.9072	0.4764	0.3114	0.031*	
H13C	0.9384	0.4806	0.4060	0.031*	

### Atomic displacement parameters (Å^2^)

**Table:**

	*U*^11^	*U*^22^	*U*^33^	*U*^12^	*U*^13^	*U*^23^
Cl1	0.0144 (2)	0.0191 (2)	0.01791 (19)	−0.00141 (14)	−0.00107 (14)	−0.00348 (12)
O1	0.0198 (6)	0.0158 (5)	0.0415 (6)	−0.0027 (5)	−0.0034 (5)	0.0074 (5)
N1	0.0120 (6)	0.0124 (5)	0.0138 (5)	0.0001 (5)	−0.0011 (5)	−0.0007 (4)
C1	0.0140 (7)	0.0103 (6)	0.0148 (6)	0.0020 (5)	0.0018 (6)	−0.0002 (5)
C2	0.0150 (8)	0.0120 (6)	0.0197 (7)	0.0007 (6)	0.0010 (6)	−0.0012 (5)
C3	0.0186 (8)	0.0133 (6)	0.0211 (7)	0.0005 (6)	0.0071 (6)	0.0020 (5)
C4	0.0247 (9)	0.0154 (6)	0.0153 (6)	0.0041 (6)	0.0035 (7)	0.0023 (5)
C5	0.0206 (8)	0.0171 (7)	0.0146 (6)	0.0029 (6)	−0.0016 (6)	0.0003 (5)
C6	0.0145 (7)	0.0125 (6)	0.0150 (6)	0.0022 (6)	0.0005 (6)	−0.0012 (5)
C7	0.0129 (7)	0.0127 (6)	0.0162 (6)	0.0030 (6)	−0.0006 (6)	−0.0019 (5)
C8	0.0168 (8)	0.0247 (8)	0.0162 (6)	−0.0040 (7)	−0.0024 (6)	−0.0006 (6)
C9	0.0124 (8)	0.0121 (6)	0.0157 (6)	0.0014 (5)	0.0001 (6)	−0.0006 (5)
C10	0.0122 (7)	0.0111 (6)	0.0142 (6)	0.0020 (6)	−0.0004 (5)	−0.0004 (5)
C11	0.0168 (8)	0.0177 (7)	0.0132 (6)	−0.0016 (6)	−0.0005 (6)	0.0008 (5)
C12	0.0160 (8)	0.0174 (7)	0.0131 (6)	−0.0038 (6)	−0.0033 (6)	−0.0005 (5)
C13	0.0173 (8)	0.0192 (7)	0.0249 (7)	−0.0023 (6)	0.0036 (7)	0.0023 (6)

### Geometric parameters (Å, º)

**Table:**

O1—C12	1.2119 (18)	C7—C9	1.3821 (18)
N1—C10	1.3188 (19)	C7—C8	1.502 (2)
N1—C1	1.3769 (17)	C8—H8A	0.9800
N1—H1	0.91 (2)	C8—H8B	0.9800
C1—C2	1.405 (2)	C8—H8C	0.9800
C1—C6	1.408 (2)	C9—C10	1.415 (2)
C2—C3	1.3762 (19)	C9—C12	1.517 (2)
C2—H2	0.9500	C10—C11	1.4894 (18)
C3—C4	1.400 (2)	C11—H11A	0.9800
C3—H3	0.9500	C11—H11B	0.9800
C4—C5	1.367 (2)	C11—H11C	0.9800
C4—H4	0.9500	C12—C13	1.486 (2)
C5—C6	1.4228 (18)	C13—H13A	0.9800
C5—H5	0.9500	C13—H13B	0.9800
C6—C7	1.426 (2)	C13—H13C	0.9800
			
C10—N1—C1	123.63 (13)	H8A—C8—H8B	109.5
C10—N1—H1	120.0 (12)	C7—C8—H8C	109.5
C1—N1—H1	116.4 (12)	H8A—C8—H8C	109.5
N1—C1—C2	119.28 (13)	H8B—C8—H8C	109.5
N1—C1—C6	118.85 (13)	C7—C9—C10	120.84 (13)
C2—C1—C6	121.86 (12)	C7—C9—C12	121.31 (13)
C3—C2—C1	118.51 (14)	C10—C9—C12	117.68 (12)
C3—C2—H2	120.7	N1—C10—C9	119.01 (12)
C1—C2—H2	120.7	N1—C10—C11	118.37 (12)
C2—C3—C4	120.69 (14)	C9—C10—C11	122.61 (13)
C2—C3—H3	119.7	C10—C11—H11A	109.5
C4—C3—H3	119.7	C10—C11—H11B	109.5
C5—C4—C3	121.10 (13)	H11A—C11—H11B	109.5
C5—C4—H4	119.4	C10—C11—H11C	109.5
C3—C4—H4	119.4	H11A—C11—H11C	109.5
C4—C5—C6	120.19 (14)	H11B—C11—H11C	109.5
C4—C5—H5	119.9	O1—C12—C13	122.82 (14)
C6—C5—H5	119.9	O1—C12—C9	118.68 (14)
C1—C6—C7	118.97 (12)	C13—C12—C9	118.47 (12)
C1—C6—C5	117.56 (13)	C12—C13—H13A	109.5
C7—C6—C5	123.42 (13)	C12—C13—H13B	109.5
C9—C7—C6	118.56 (13)	H13A—C13—H13B	109.5
C9—C7—C8	122.14 (13)	C12—C13—H13C	109.5
C6—C7—C8	119.21 (12)	H13A—C13—H13C	109.5
C7—C8—H8A	109.5	H13B—C13—H13C	109.5
C7—C8—H8B	109.5		
			
C10—N1—C1—C2	177.60 (13)	C5—C6—C7—C8	−3.1 (2)
C10—N1—C1—C6	−1.1 (2)	C6—C7—C9—C10	−0.9 (2)
N1—C1—C2—C3	−178.66 (12)	C8—C7—C9—C10	−177.57 (13)
C6—C1—C2—C3	0.0 (2)	C6—C7—C9—C12	174.34 (13)
C1—C2—C3—C4	−2.4 (2)	C8—C7—C9—C12	−2.3 (2)
C2—C3—C4—C5	2.5 (2)	C1—N1—C10—C9	−2.3 (2)
C3—C4—C5—C6	−0.1 (2)	C1—N1—C10—C11	176.77 (12)
N1—C1—C6—C7	3.45 (19)	C7—C9—C10—N1	3.3 (2)
C2—C1—C6—C7	−175.19 (13)	C12—C9—C10—N1	−172.12 (12)
N1—C1—C6—C5	−179.02 (12)	C7—C9—C10—C11	−175.71 (13)
C2—C1—C6—C5	2.3 (2)	C12—C9—C10—C11	8.8 (2)
C4—C5—C6—C1	−2.3 (2)	C7—C9—C12—O1	−101.78 (17)
C4—C5—C6—C7	175.13 (13)	C10—C9—C12—O1	73.64 (18)
C1—C6—C7—C9	−2.4 (2)	C7—C9—C12—C13	80.14 (17)
C5—C6—C7—C9	−179.80 (13)	C10—C9—C12—C13	−104.45 (15)
C1—C6—C7—C8	174.32 (13)		

### Hydrogen-bond geometry (Å, º)

**Table:**

*D*—H···*A*	*D*—H	H···*A*	*D*···*A*	*D*—H···*A*
N1—H1···Cl1	0.91 (2)	2.13 (2)	3.0374 (13)	175.4 (17)
